# ^1^H Time Domain Nuclear Magnetic Resonance and Oscillatory Rheology as a Tool for Uncovering the Impact of UV-C Radiation on Polypropylene

**DOI:** 10.3390/polym17202727

**Published:** 2025-10-11

**Authors:** Jessica Caroline Ferreira Gimenez, Sophia Helena Felisbino Bonatti, Marcos Vinícius Basaglia, Rodrigo Henrique dos Santos Garcia, Alef dos Santos, Lucas Henrique Staffa, Mazen Samara, Silvia Helena Prado Bettini, Eduardo Ribeiro de Azevedo, Emna Helal, Nicole Raymonde Demarquette, Manoel Gustavo Petrucelli Homem, Sandra Andrea Cruz

**Affiliations:** 1 Department of Chemistry, Exact Sciences and Technology Center (CCET), Federal University of São Carlos (UFSCar), Rodovia Washington Luís, Km 235, SP-310, São Carlos 13565-905, SP, Brazil; jessicagimenez@estudante.ufscar.br (J.C.F.G.); sophiabonatti@estudante.ufscar.br (S.H.F.B.); alefsantos122@gmail.com (A.d.S.); mghomem@ufscar.br (M.G.P.H.); 2 Department of Mechanical Engineering, École de Technologie Supérieure (ÉTS), 1100 Notre-Dame St W, Montréal, QC H3C 1K3, Canada; mazen.samara@etsmtl.ca (M.S.); emna.helal@etsmtl.ca (E.H.); nicoler.demarquette@etsmtl.ca (N.R.D.); 3 Department of Materials Engineering, Exact Sciences and Technology Center (CCET), Federal University of São Carlos (UFSCar), Rodovia Washington Luís, Km 235, SP-310, São Carlos 13565-905, SP, Brazil; marcosbasaglia@estudante.ufscar.br (M.V.B.); lucas.staffa@ufscar.br (L.H.S.); silvia.bettini@ufscar.br (S.H.P.B.); 4 Physics Institute of São Carlos, IFSC, University of São Paulo, Avenida Trabalhador Sãocarlense, 400, São Carlos 13566-590, SP, Brazil; rodrigogarcia@alumni.usp.br (R.H.d.S.G.); azevedo@ifsc.usp.br (E.R.d.A.); 5 NanoXplore Inc., 4500 Thimens Blvd, Saint-Laurent, Montréal, QC H4R 2P2, Canada

**Keywords:** UV-C, polypropylene photodegradation, rheology, ^1^H Time Domain Nuclear Magnetic Resonance

## Abstract

UV-C radiation has emerged as a germicidal agent against pathogens, particularly following the COVID-19 pandemic. While UV-C effectively reduces cross-contamination in hospitals, it induces photodegradation in polymer devices, potentially damaging and posing risks to patient safety. Therefore, it is crucial to detect the effects of UV-C photodegradation on early stages, as well as the effects of prolonged UV-C exposure. In this study, we investigated the UV-C photodegradation (254 nm, 471 kJ/mol) of isotactic polypropylene homopolymer (PP), commonly used in medication packaging. The impact of UV-C on PP was evaluated through rheology and infrared spectroscopy. Surface energy was measured by the contact angles formed by drops of water and diiodomethane. The effects of photodegradation on the polymer’s morphology were examined using scanning electron microscopy, and the melting temperature and crystallinity by differential scanning calorimetry. Lastly, the effect of UV-C on molecular mobility was studied using ^1^H Time Domain Nuclear Magnetic Resonance (^1^H TD-NMR). These techniques proved to be valuable tools for identifying the early stages of UV-C photodegradation, and ^1^H TD-NMR was a sensitive method to identify the chain branching as a photodegradation product. This study highlights the impact of UV-C on PP photodegradation and hence the importance of understanding UV-C-induced degradation.

## 1. Introduction

The use of germicidal lamps emitting ultraviolet light in the 100–280 nm wavelength range (UV-C) gained prominence during the COVID-19 pandemic [1,2,3,4,5,6]. UV-C radiation is widely employed for sterilizing medical facilities and various public and private spaces, helping to prevent the spread of viruses and other pathogens, particularly by reducing the risk of cross-contamination [7]. Characterized as the most energetic within the UV spectrum, UV-C traditionally straddles the boundary between ionizing and non-ionizing radiation [8]. While its high energy makes it capable of disrupting nucleic acids (DNA), it also has the potential to break chemical bonds in materials such as wood, paper, and polymers [9].

Plastic devices, particularly those produced from polypropylene (PP), are extensively employed not only in medical settings, but also in various household appliances and consumer goods. PP homopolymer, valued for its versatility, finds applications in various medical tools, including syringes, drug delivery systems, fabrics, tubes, personal protective equipment, and packaging for medication [10,11], as well as in food packaging, tapes, films, bottles, and as a polymer matrix in composite materials [12,13]. However, the susceptibility of PP products to ultraviolet light limits their efficacy in medical environments. In principle, polyolefins such as polypropylene consist solely of C-C and C-H bonds and are not expected to absorb light at wavelengths above 200 nm. Nevertheless, due to the polymerization process and subsequent processing steps, certain chromophore groups or impurities, including traces of catalysts and additives, may be present in the polymer’s structure, leading to the photodegradation of the material [14].

The photodegradation mechanism of PP under UV-A and UV-B is well-documented in the literature [15,16,17,18,19,20,21,22,23,24]. It starts when these chromophore groups absorb UV energy and form charge-transfer (CT) complexes [14]. These CT complexes initiate the photodegradation process by homolytic scission of the PP chain, generating free radicals that attack the neighboring polymer chains. In the presence of oxygen, this series of events triggers a self-auto-oxidation cycle known as photooxidation, resulting in chromophore groups such as carbonyls and double bonds in the polymer chain [15,16,17,18,19,20,21,22,23,24,25]. In the photodegradation of polypropylene (PP) under UV-A and UV-B radiation, chain scission reactions predominate over crosslinking and branching reactions [26], decreasing the molar mass and causing significant alterations in physicochemical properties.

Extensive research has examined the effects of UV-A and UV-B radiation on polymers [15,16,17,18,19,20,21,22,23,24]. However, despite the widespread use of UV-C radiation for disinfecting tools, equipment, and surfaces, particularly in medical facilities, few studies [27,28] have focused on understanding the degradation processes induced by this higher-energy radiation. This gap is critical, as the durability of different materials under UV-C exposure, specifically, which materials can withstand it, which cannot, and the thresholds of their resistance, remains largely unresolved. Therefore, it is essential to identify the early stages of UV-C-induced photodegradation and to understand how prolonged UV-C exposure affects materials in order to develop more effective strategies for their protection.

Regarding sterilization with UV-C light in medical plastic devices, two critical aspects of photodegradation are often overlooked: early-stage detection and evaluation after prolonged exposure. The absence of methodologies to investigate photodegradation and protocols to detect its early stages in polymers used in medical devices can directly impact the equipment’s lifespan and endanger patient safety. For instance, Irving et al. [28] reported that UV-C exposure compromised the structural integrity of flexible endoscope tubes. After 400 h of exposure to UV-C light at 245 nm, the material exhibited increased wettability, surface cracks, and chain scission at the molecular level.

Basaglia et al. [29]. investigated the effect of photostabilizers on UV-C photoprotection in polypropylene. The authors evaluated the impact of various photostabilizers on carbonyl formation and PP mechanical properties, including phenolic antioxidants, hydroxylamines, and UV absorbers. Although the photostabilizing system significantly suppressed carbonyl formation, the samples became brittle after 96 h of UV-C exposure. The study also demonstrated that combining hydroxylamines or phenolic antioxidants with UV absorbers resulted in a synergistic effect, preserving the ductility of polypropylene and allowing deformations of over 300% without fractures.

To the best of our knowledge, no studies in the literature focus on elucidating the effect of UV-C photodegradation on polypropylene or providing a new, solvent-free, reliable approach for differentiating crosslinking and branching byproducts.

In this context, ^1^H Time Domain Nuclear Magnetic Resonance (^1^H TD-NMR) is a solvent-free technique that has recently been gaining prominence in evaluating degradation processes in polymers [30,31,32,33]. Although ^1^H TD-NMR is particularly effective for investigating the microstructure, mobility, and phase morphology of polymers [30,31,32,34,35], this methodology has proven reliable in identifying photodegradation byproducts in polymers. By detecting the signal intensity related to double quantum coherences between ^1^H nuclei spins $\left(I_{DQ}\right)$ induced by the presence of ^1^H-^1^H dipolar magnetic coupling, ^1^H DQ-TDNMR can provide information about chemical and physical constraints present in polymer matrices. Furthermore, this technique can also quantify the fraction of free mobile (unconstrained) chains, making it a powerful tool for studying phenomena such as crosslinking, branching, and chain entanglements [36].

Therefore, in light of the challenges identified, this study aims to address these issues by: (i) identifying early-stage degradation processes, (ii) performing a temporal evaluation of degradation mechanisms, especially after prolonged exposure, and (iii) using a reliable, solvent-free method to distinguish crosslinking and branching photodegradation byproducts in polypropylene, to thereby, provide a better understanding and to elucidate the UV-C effect on polymer photodegradation, providing highlights to properly photostabilize and enhance its UV-C resistance.

Thus, the effect of UV-C exposure on polypropylene (PP) samples was investigated using a combination of analytical techniques, including FTIR-ATR, rheology, measurement of water contact angle formed by water droplets on the exposed surface, DSC, and ^1^H TD-NMR. Some of these analyses allowed the detection of early signs of degradation during the initial stages of the process (<6 h). It was possible to identify a shift in the degradation mechanism: from chain scission, which leads to a reduction in molar mass in the early stages, to an increase in molar mass, suggesting branching or crosslinking, in more advanced stages of photodegradation. The ^1^H TD-NMR technique, in particular, enabled distinguishing between these two processes, indicating that branching is the predominant mechanism in this case. This comprehensive approach effectively enables the identification and understanding of UV-C radiation-induced degradation in polypropylene, providing valuable insights into the material’s behavior under prolonged exposure.

## 2. Materials and Methods

### 2.1. Materials and Reagents

The homopolymer PP pellets used in this work were HP 523J grade, supplied by Braskem with a melt flow index (MFI) of 3.1 g/10 min (Standard ASTM 1238 [37], 230 °C, 2.16 kg), tensile strength at flow of 33 MPa (Standard ASTM D638 [38]), elongation in flow of 9% (ASTM D638), Izod impact Strength at 23 °C of 30 J/m (Standard ASTM D256 [39]), and a density of 0.902 g/cm^3^ (Standard ASTM D792 [40]). This grade is used for the extrusion of bi-oriented films that can be employed as an overlay for medication packaging. The PP pellets were extruded once using a Thermo Fisher Process 11 Parallel Twin-Screw Extruder (ThermoFisher, USA) at 200 °C along the barrel, with a screw speed of 100 rpm. The extruded PP was then hot-pressed using a TIL MARCON MPH-10 hydraulic press (MARCON, Brazil), operated at 200 °C and 0.05 tons of pressure, to produce film samples with a thickness of approximately 0.1 to 0.2 mm, and a diameter of approximately 6 cm. The PP pellets were heated, hot-pressed, and then cooled under water at room temperature to produce thin films. The PP pellets underwent no pretreatment prior to processing.

### 2.2. UV-C Chamber Assembly

PP films were exposed to a custom-built UV-C metallic chamber with a 33 cm diameter and 29 cm height (Figure S1-supplementary). A fan was installed on one side to ensure complete ozone removal. The UV-C source used consisted of two commercial Hg lamps (Philips, Brazil, TUV 4 W) emitting light at a peak wavelength of 254 nm (471 kJ/mol). These lamps, measuring 15 cm in length and 1.60 cm in diameter, were positioned 26 cm apart. The sample holder was placed between them to ensure exposure of the samples from both sides. The irradiation intensity on the sample surface was approximately 1.3 mW/cm^2^. The exposure times were set at 0, 1, 2, 4, 6, 12, 24, 48, 96, 192, and 384 h. Subsequently, the samples were promptly analyzed.

### 2.3. Physicochemical Analysis

Rheological characterization of the photodegraded samples was performed using an Anton Paar MCR 302 rheometer (Anton Paar, Austria). Small amplitude oscillatory shear tests were employed over a frequency range of 0.01 to 500 rad·s^−1^, and using a 25 mm parallel plate geometry with a 1 mm gap at 200 °C. These experiments were performed at 3% strain, within the linear viscoelastic region, which was established from an amplitude sweep test at 200 °C with an angular frequency of 1 rad·s^−1^ and a strain range of 0.01–100%. Time sweep experiments were performed at a frequency of 1 Hz to evaluate the stability of the samples. All the experiments were performed in a nitrogen atmosphere. The samples were analyzed in duplicate.

^1^H TD-NMR experiments were performed in a 0.5-T Bruker Minispec mq20 (Bruker, USA) NMR analyzer (^1^H frequency of 20 MHz) using a VT probe head with a dead time of 11.5 μs. π/2 and π pulse lengths of 2.5 and 4.8 µs, respectively. The longitudinal relaxation (T_1_) of the samples was measured using a standard Inversion-Recovery pulse sequence. The recycling delays of all experiments were adjusted to 5T_1_. Mixed-Magic Sandwich Echo Experiments (mixed-MSE) were acquired with an echo time of 100 ms. Dipolar Filtered MSE (DF-MSE) experiments were also performed with echo times of 100 μs and dipolar filter time of 40 μs at temperatures varying from 303 to 463 K. The pulse sequence and phase cycling used in these experiments are described in reference [34]. ^1^H DQ-TDNMR experiments used double quantum evolution times ranging from 0.1 to 14.3 ms. The data were acquired using the pulse sequence and phase cycling described in reference [35]. ^1^H DQ-TDNMR measurements were performed at 190 °C, above PP’s melting temperature. More details about this– technique can be found in the Supplementary Materials section. All NMR experiments were performed twice, yielding identical results. To provide a more representative sampling, five pieces of 8 mm diameter were taken from different regions of the irradiated films, cut, and combined in a single NMR tube.

FTIR spectra of the exposed samples were obtained using an Attenuated Total Reflectance (ATR) accessory in a Thermo Scientific Nicolet 6700-FTIR spectrometer, (ThermoFisher, USA), with a wavenumber ranging from 400 to 4000 cm^−1^, using 64 scans and a resolution of 4 cm^−1^. The samples were analyzed in triplicate.

SEM micrographs of the exposed sample surfaces were obtained using an FEI Inspect S50 microscope (FEI Company-ThermoFisher, USA) at 10 kV. All samples were coated with a Au coating by sputtering.

Before and after UV-C exposure, contact angles formed by drops of water and diiodomethane were measured using a Ramé-hart 260-F4 Series goniometer (Ramé-hart, USA). For polymeric surfaces, the surface energy was evaluated by the harmonic mean equation proposed (Equation (1)) by Wu [41]:

(1) $$\gamma_{LV}\left(1+\cos\theta \right)=4\left[\frac{\gamma_{SV}^{D}\times \gamma_{LV}^{D}}{\gamma_{SV}^{D}+\gamma_{LV}^{D}}+\frac{\gamma_{SV}^{P}\times \gamma_{LV}^{P}}{\gamma_{SV}^{P}+\gamma_{LV}^{P}}\right]$$

where $\gamma_{LV}$ is the liquid/vapor component, θ is the angle formed by the drops, $\gamma_{SV}^{D}$ and $\gamma_{LV}^{D}$ are the dispersive components for solid/vapor and liquid/vapor, respectively, and $\gamma_{SV}^{P}$ and $\gamma_{LV}^{P}$ are the polar components for solid/vapor and liquid/vapor. Each sample was analyzed in triplicate, employing three droplets of water and diiodomethane for each replicate.

A Netzsch DSC 200 F3 (Netzsch, Germany) was used to perform differential scanning calorimetry (DSC). Samples weighing ~10.0 mg were heated from 20 °C to 210 °C at a heating rate of 10 °C/min under a nitrogen gas flow. The degree of crystallinity (χ) of all samples was calculated following Equation (2), where ${\Delta H}_{f}$ refers to the melting enthalpy and ${\Delta H}_{f}^{{}^{\circ}}$ denotes the standard heat of melting of a hypothetically 100% crystalline PP, which was used 209 J/g [42]. The samples were analyzed in duplicate.

(2) $$\chi =\frac{{\Delta H}_{f}}{{\Delta H}_{f}^{{}^{\circ}}}\times 100\%$$

## 3. Results

### 3.1. Rheological and ^1^H DQ-TDNMR Analyses for the Samples

**Figure 1:**
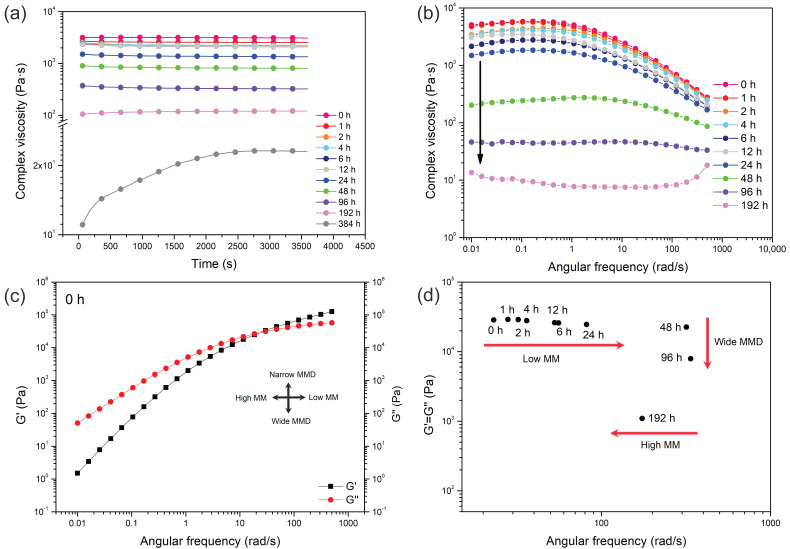
(**a**) Complex viscosity as a function of time for all samples, (**b**) complex viscosity as a function of frequency for all samples up to 192 h, (**c**) loss and storage moduli for PP at 0 h, and (**d**) cross-over point for all samples up to 192 h.

Figure 1a presents the results of complex viscosity as a function of time obtained during time sweep experiments for PP samples at different UV-C exposure times. A complete explanation of the rheological concepts used to analyze the following samples is provided in the Supplementary section. An unexpected behavior was observed in the sample exposed for 384 h, referred to as sample 384, which exhibited significant instability during the time sweep test, with an increase in viscosity. This behavior may be attributed to the recombination of free radicals generated during prolonged exposure to high-energy UV-C radiation (approximately 471 kJ/mol at 254 nm) [43]. These free radicals may recombine into higher molar mass species through crosslinking or branching, leading to an increase in viscosity.

Figure 1b presents the complex viscosity as a function of frequency for all samples except for that exposed for sample 384, since it exhibited instability. As expected, the PP viscosity decreases upon UV-C exposure, and samples exposed to more than 48 h of UV-C showed a less pronounced pseudoplastic behavior, indicating a possible change in the molar mass distribution (MMD). A solubility test in xylene at 140 °C was performed before and after the rheological tests to investigate whether the increase in viscosity in sample 384 was due to crosslinking or branching. The absence of gel formation indicated that the increase in viscosity was likely caused by branching.

Cross-over points in the rheological data, where G′(ω) equals G″(ω), as shown in Figure 1c, provide insights into the evolution of molar mass (MM) and its distribution (MMD), as described by Cruz et al. [44]. Figure 1d shows the crossover points for all the samples studied in this work. The results indicate that for samples exposed from 0 h to 96 h, there is a reduction in the molar mass. After 96 h of exposure, a shift occurs, indicating that some compounds with higher molar mass may have formed, along with a broadening in the molar mass distribution. This indicates a change in the mechanism of UV-C photodegradation at higher times. G′(ω) and G″(ω) curves can be found in the Supplementary Materials (Figure S2).

Studies found in the literature showing the rheological behavior resulting from photodegradation upon exposure to UV-A and UV-B are quite different from that observed upon exposure to UV-C employed in this study, as shown in Figure 1a. We attribute this to the fact that UV-C corresponds to a more energetic wavelength than UV-A and UV-B, which results in a higher PP photodegradation rate. Further studies are needed to clarify whether a more extended exposure to UV-A or UV-B affects PP in a similar way to when it is exposed to UV-C for a short time. This was not considered within the scope of this study.

**Figure 2:**
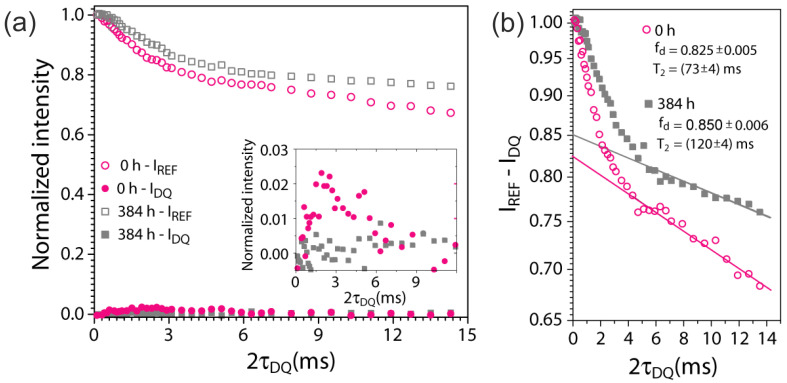
(**a**) Normalized double quantum and reference intensities as a function of $\tau_{DQ}$ for PP and PP with 384 h exposure to UV-C radiation, measured at 190 °C, (**b**) the $I_{REF}-I_{DQ}$. $vs\;\tau_{DQ}$ curves in logarithmic scale and the exponential fit curves used to extract the fraction of free mobile chains *f*_d_ and the corresponding T_2_ relaxation times.

Figure 2a shows the normalized double quantum and reference intensities associated with all ^1^H spins in the sample $\left(I_{REF}\right)$ and the intensity related to the so-called double quantum coherences between ^1^H nuclear spins $\left(I_{DQ}\right)$. This intensity is induced by the presence of ^1^H—^1^H dipolar magnetic coupling between ^1^H nuclei in constrained regions of the samples. Since double quantum coherence needs are only created if ^1^H—^1^H dipolar magnetic coupling is present, the buildup of this intensity as a function of the evolution period $(\tau_{DQ}$) means that there are local mobility constraints, which in the melt state are intimately associated with chain entanglement or crosslinks. The $I_{DQ}$ and $I_{REF}$ curves as a function of the evolution period $(\tau_{DQ}$) at 190 °C (above the PP melting temperature) for the samples with 0 and 384 h of UV-C exposure are shown in Figure 2a. The curves were normalized by the initial point of the corresponding $I_{REF}\;vs\;\tau_{DQ}\;$ curve. Figure 2b illustrates the $I_{REF}-I_{DQ}$ and the fitting of the long 2$\tau_{DQ}$ region of the curves for both samples.

For the non-exposed sample, 0 h, a rather small *I_DQ_* intensity is observed, which drops for the sample 384. The drop in *I_DQ_* indicates an increase in the fraction of unconstrained molecular segments shown in the inset in Figure 2a.

Figure 2b illustrates the $I_{REF}-I_{DQ}$ and the fitting of the long 2$\tau_{DQ}$ region of the curves for both samples. As can be seen, there is an increase in the fraction of unconstrained molecular segments (*f*_d_) and the corresponding relaxation time (*T*_2_). This indicates that while there is an increase in the free mobile chain, their overall mobility is also enhanced. Thus, the increase in the fraction of free mobile chains and the absence of $I_{DQ}$ intensity, shown in the inset in Figure 2a, in samples exposed to UV-C, suggest that, while exposure to UV-C radiation induces free mobile chains, it does not produce a detectable fraction of crosslinks. Instead, it decreases the fraction of the entangled chains. Thus, the decrease in the signal, which is related to fewer entanglements, shows that the polymer increases its molar mass through branching rather than crosslinking.

To evaluate the branching reactions, the increase in the relaxation time *T*_2_ for the sample after 384 h, as retrieved from the curves shown in Figure 2, was used. The increase in the fraction of free mobile chains is in line with the rheological analysis (Figure 1), as it can also result from chain branching caused by degradation processes at higher times of exposure to UV-C light. More interestingly, it suggests that the molar mass increase is likely due to chain branching rather than crosslinking.

### 3.2. Effect of UV-C Light on the Molecular Mobility and Microstructure

**Figure 3:**
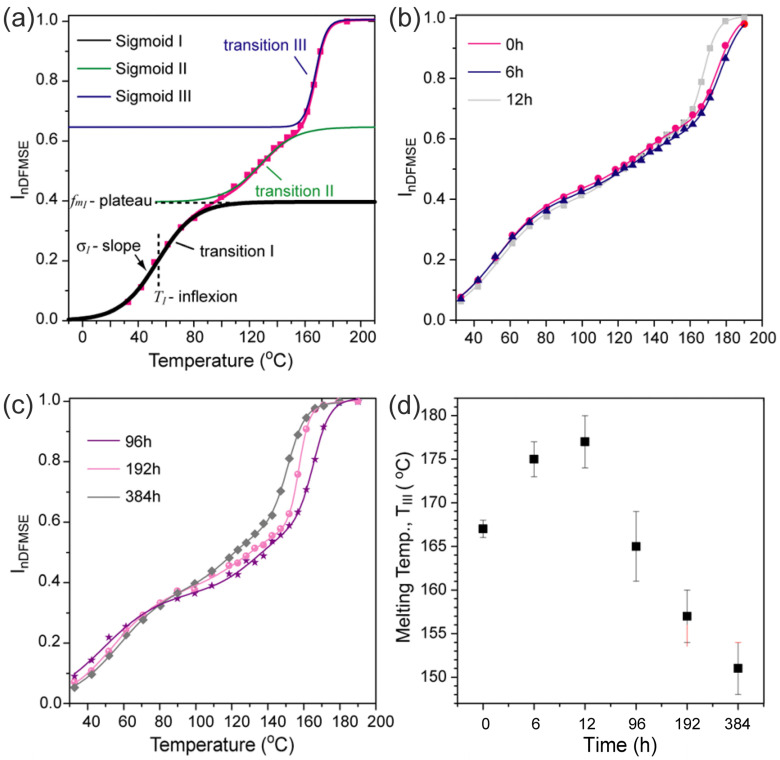
(**a**) $I_{nDFMSE}\;vs.\;T$ curve obtained for the sample PP 0 h and the corresponding fit using N = 3 components, sum of a weighted sigmodal function. For better visualization, the curves for sigmoids II and III were shifted up using the corresponding fractions. (**b**) Comparison of $I_{nDFMSE}\;vs.\;T$ curves obtained for samples PP 0 h, PP 6 h, and PP 12 h. (**c**) Same as (**b**) for samples PP 96 h, PP 192 h, and PP 384 h. (**d**) Melting temperatures ($T_{III}$) of all analyzed samples obtained from the fit of the $I_{nDFMSE}\;vs.\;\;T$ curves. In (**a**–**c**), the intensity errors are smaller than the points.

The dipolar filtered magic Sandwich Echo (DF-MSE) intensity as a function of temperature ($I_{nDFMSE}\;vs.\;T\;$ curve), obtained for the not exposed PP sample, is shown in Figure 3a. ^1^H time-domain measurements at a low magnetic field were used to investigate changes in molecular mobility due to UV irradiation. This was achieved using the so-called Dipolar Filtered Magic Sandwich Echo (DF-MSE) experiment, which is a solid-state NMR technique that filters out signals from rigid segments, so that the measured intensity, $I_{nDFMSE}$, reflects only the ^1^H signal from molecular segments that are mobile. For more details, see the work of Filgueiras et al. [45] and Perez et al. [32]. In this context, rigid and mobile segments are defined according to whether the molecular dynamics are fast enough to reach a critical frequency capable of averaging out the strong dipolar interaction between ^1^H nuclei, which drastically modifies the detected NMR signal.

By monitoring $I_{nDFMSE}$ as a function of temperature, the signal remains negligible while molecular motions are below the critical frequency. As the temperature increases and the motions reach this critical frequency, $I_{nDFMSE}$ rises, marking the onset of detectable mobility. For a given transition i, this produces typical curves as a function of temperature, $I_{nDFMSE}\;vs.\;T\;$, with an “S”-shaped profile, as represented by the black line represented in Figure 3a. These curves can be phenomenologically described by sigmoidal functions: the inflection temperature defines the transition temperature ($T_{i})$; the plateau height reflects the fraction of mobile segments involved in the transition ($f_{mi})$, and the slope ($\sigma_{i})$, indicates whether the transition is sharp or gradual, thereby providing insight into dynamic heterogeneity (greater heterogeneity corresponds to higher $\sigma_{i}$). These parameters are illustrated in Figure 3a, and the equation for the sigmoidal curve is:

(3) $$I_{nDF-MSE}\left(T\right)=\frac{f_{mi}}{1+e^{-\left(T-T_{i}\right)/\sigma_{i}}}$$

In systems undergoing multiple dynamic transitions, the $I_{nDFMSE}\;vs.\;T\;$ curve can be understood as the superposition of several “S”-shaped contributions, each associated with a fraction of segments gaining mobility within a given temperature range. This allows not only the identification of the corresponding inflection temperatures but also the fraction of segments involved in each process. As an example, The $I_{nDFMSE}\;vs.\;T\;$ curve obtained for the non-exposed PP sample, is shown in Figure 3a.

Three intensity increases associated with the onset of segmental motions were identified in Figure 3a. The first (transition I, 20–80 °C) corresponds to the glass transition. The second (transition II, 100–150 °C) is attributed to pre-melting molecular relaxations in PP (helical jumps) [46,47] and to motions of constrained amorphous chains at the crystalline–amorphous interphase. The third (transition III, 150–200 °C) reflects the onset of segmental motions induced by melting of the crystalline phase. A three-component version of Equation (3) was used to fit the experimental $I_{nDFMSE}\;vs.\;T$ curve to obtain $f_{mi}$, $T_{i}$ and $\sigma_{i}$ for each transition. The best-fitting sigmoidal curve, as well as the three components, are shown separately in Figure 3a. The parameters extracted from fitting the $I_{nDFMSE}\;vs.\;T$ curve are shown in Table 1.

The effect of UV-C irradiation on the $I_{nDFMSE}\;vs.T$ curves are shown in Figure 3b for short exposure times (6 and 12 h) and in Figure 3c for longer exposures (96, 192, and 384 h). At short times, only a slight increase in the melting-related process temperature (transition III) is observed (Figure 3b). Indeed, except for $T_{III}$, all curves can be fitted with the same $f_{mi}$, $T_{i}$ and $\sigma_{i}$ within experimental error, as summarized in Table 1. However, at longer times, Figure 3c reveals a clear shift in transition III, with $T_{III}$ progressively decreasing as exposure increases. This trend is highlighted in Figure 3d, which plots the fitted $T_{III}$ values as a function of irradiation time.

Table 1 presents the full set of parameters obtained from the fittings of the $I_{nDFMSE}\;vs.\;T\;$ curves. Since each transition involves three parameters, the fitting procedure carries relatively large uncertainties in the fractions and widths of the sigmoidal curves. Nevertheless, the transition temperatures could be determined with good accuracy.

As shown in Table 1, $T_{I}$ and $T_{II}$ remained essentially unaffected by UV-C irradiation, whereas pronounced changes were observed in $T_{III}$ after 96, 192, and 384 h of exposure. The decrease in $T_{III}$ reflects modifications in the melting behavior of the crystalline phase. The values of $\sigma_{I}$ and $\sigma_{II}$ remained nearly constant and higher than $\sigma_{III}$, consistent with transition III being associated with melting. The fraction of ^1^H in the segments $f_{mi}$ was unchanged across all samples.

**Figure 4:**
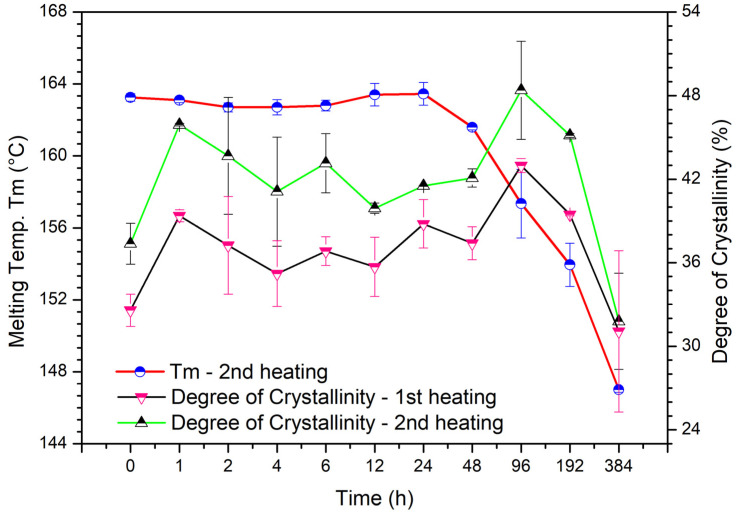
Melting temperature (T_m_) and degree of crystallinity for all irradiated samples by UV-C.

Figure 4 illustrates the impact of UV-C on crystallinity and melting temperature for all samples measured by DSC, including the samples not exposed to UV-C. Table S1 in the Supplementary section presents the values obtained from the DSC analysis, and Figure S3, also in the in the Supplementary section, presents the thermogram of the second heating for the melting temperature. For the first and second heating, no changes in crystallinity were observed within the first 96 h. However, beyond this period, a reduction in the degree of crystallinity became evident.

According to the literature, exposure to UV-A and UV-B typically increases crystallinity through chemo-crystallization [48,49]. Notably, such phenomena were not observed with UV-C, as shown in Figure 4, which explains no crack formation. Additionally, crosslinking and branching processes may hinder polymer reorganization into crystals, decreasing the degree of crystallinity.

Due to the inherent nature of the polymer, a high concentration of entanglements is expected, impacting both the melting temperature and the crystallization process. The peak melting temperature (T_m_) during the second heating cycle is also shown in Figure 3. Prior to 48 h of irradiation, the T_m_ remained stable at approximately 163 °C. However, after 96, 192, and 384 h under UV-C exposure, the temperature decreased by 7 to 16 °C. Unlike crosslinked chains, where T_m_ tends to increase, branched chains impact the free volume between polymer chains, enhancing the mobility of smaller molecules resulting from chain scission and subsequently reducing T_m_. This trend is also observed in the $T_{III}\;({}^{\circ}C)$ values provided in Table 1. Therefore, chain branching probably prevails over crosslinking, especially with prolonged exposure to UV-C, potentially leading to a decrease in crystallinity and the peak melting temperature.

### 3.3. Chemical and Morphological Alterations for the Samples

**Figure 5:**
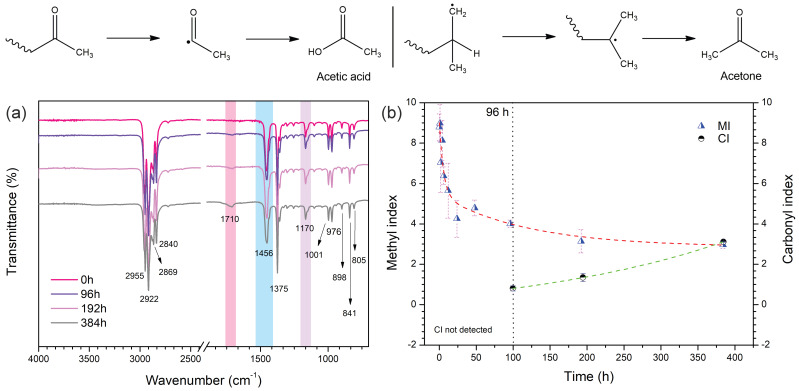
(**a**) FTIR spectra for PP exposure to 0, 96, 192, and 384 h, and (**b**) methyl and carbonyl indices for all samples.

Figure 5a shows the FTIR spectra of PP exposed to UV-C for 0, 96, 192, and 384 h, while Figure 5b presents methyl (MI) and carbonyl (CI) indices as a function of UV-C exposure time. The vibration type and assignment for pristine PP are provided in the Supplementary Material (Table S2). It was used the methyl peak at 1456 cm^−1^, which corresponds to asymmetric bending in the plane of the C—H bond in CH_3_ moieties [50] for the MI, and the carbonyl peak, assigned to around 1710 cm^−1^/1735 cm^−1^ [16,50,51], was used for CI. The main chain’s stretching of the C—C bond (at 1170 cm^−1^) was used as an invariant peak [52], all peaks used are highlighted in Figure 5 (a). This peak exhibits a shoulder at 1153 cm^−1^, which is assigned to the CH_3_ wagging of the regular head-to-tail sequence of isotactic polypropylene [53,54]. The indices were calculated based on the area of the deconvoluted peak using the Voigt function.

The results depicted in Figure 5b demonstrate that the carbonyl peak, used to calculate CI, only becomes detectable after 96 h of exposure to UV-C, as observed in Figure S4 in the Supplementary materials. Thus, using it to assess the early stages of photodegradation under UV-C may lead to inaccuracies in predicting the life span of PP devices. Concurrently, MI exhibits a noticeable decrease after less than 10 h of UV-C exposure, indicating a significantly higher sensitivity to detecting early stages of photodegradation compared to CI. This sensitivity was also observed by Roullion et al. [50], who examined the photodegradation of PP exposed to both UV-A and UV-B. They attributed this phenomenon to the release of volatile products containing CH_3_ moieties (such as acetone and acetic acid), which were detected at 25 h of irradiation (Figure 5). The results obtained in the present study indicate that volatile compounds with CH_3_ moieties are initially generated when PP is exposed to UV-C, followed by carbonyl. This finding validates the use of FTIR-ATR to study the photodegradation of PP under UV-C.

**Figure 6:**
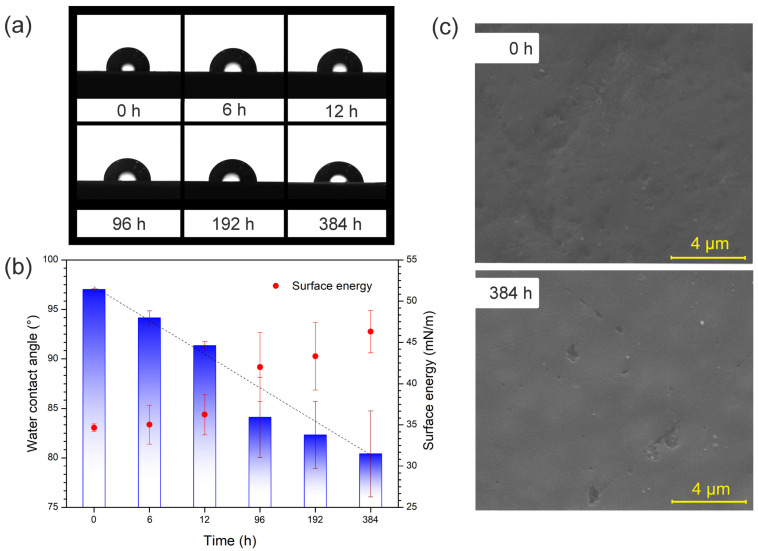
(**a**) images of the droplets of water with 6, 12, 96, 192, and 384 h of UV-C exposure; (**b**) contact angle and surface energy for samples with 0, 6, 12, 96, 192, and 384 h of irradiation by UV-C; (**c**) SEM with magnifications 20,000× for samples with 0 h and 384 h of exposure time.

Figure 6a shows images of the droplets of water with 6, 12, 96, 192, and 384 h of UV-C exposure. Figure 6b shows the changes in the contact angle and surface energy for the samples before and after 6, 12, 96, 192, and 384 h of UV-C exposure. Figure 6c shows SEM images for the samples before and after 384 h of exposure.

The contact angles formed by drops of water, shown in Figure 6a,b, decreased while the surface energy increased as a function of UV-C time exposure. The increase in surface energy, probably, indicates that the photooxidation changes the surface chemistry, impacting the hydrophilicity and wettability [48,55,56]. The contact angle technique detected changes at early stages, from 6 h on, in wettability and surface energy. The results from Figure 6b agree with the findings in the literature regarding polymer photodegradation.

The surface morphology of the sample can significantly influence the contact angle formed by water droplets; thus, a regular and smooth surface is crucial to validating the results from this analysis. The images obtained by SEM (Figure 6c) showed no cracks and no changes in the topography of sample 384; the same morphology was observed for samples with 12 h, 96 h, and 192 h of UV-C photodegradation, as shown in Figure S5 provided in the Supplementary Materials. The cross-section is also provided in the Supplementary Materials (Figure S6).

Photodegradation, as is well known, can change the polymer’s topography through the formation of cracks on the photodegraded surface [25,48,57,58]. This process normally begins with microvoids, which sequentially form cracks on the surface of the polymer due to the reduction in the sample’s volume induced by chemo-crystallization [25]. Results regarding the effect of UV-C on crystallization are detailed in the section *Effect of UV-C Light on Molecular Mobility and Microstructure*. As the cracks propagate, the surface area exposed to UV radiation increases, accelerating photo-oxidation. This leads to the formation of an affected surface layer that gradually penetrates deeper into the bulk of the material, layer by layer, resulting in cracks that are easily observed by SEM [48]. Figure S7, in the Supplementary section, shows photographs of the films taken after 0, 192, and 384 h of UV-C exposure. Over time, surface cracks became more evident, and the samples gradually became more brittle.

## 4. Discussion

**Scheme 1:**
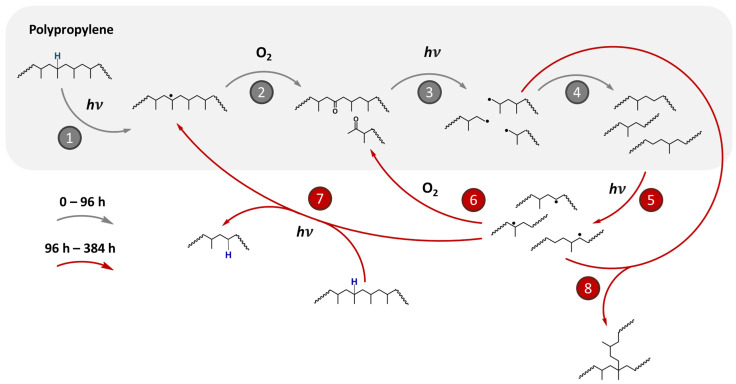
Proposed pathway of photodegradation of polypropylene under UV-C.

Scheme 1 shows the proposed pathway for UV-C photodegradation based on the experimental evidence presented in this work. This pathway can be elucidated through eight stages occurring during two intervals (before and after 96 h of exposure), as indicated by the gray and red arrows.

During the initial 96 h of exposure to UV-C (254 nm), polypropylene absorbs photons, leading to homolytic cleavage of the hydrogen-carbon bond (stage 1), resulting in the formation of a tertiary carbon radical [59]. Subsequently, this radical species undergoes an attack by an oxygen molecule, leading to chain cleavage of polypropylene and the formation of a carbonyl group (stage 2). The carbonyl group formation has been previously described by Allen et al. [59] and Rabek [14], and can be observed in the infrared spectrum and the carbonyl index after 96 h of exposure (Figure 5a,b). Additionally, in this step, form volatile products, acetic acid and acetone, both containing CH_3_ groups. Subsequently, these carbonyl products may undergo a photocatalytic reaction governed by a Norrish mechanism type [59], wherein the oxidized products absorb more photons and the carbonyl group cleavage occurs, leading to the formation of more radicals (stage 3), which can recombine into low molar mass species (stage 4). The proposed stage (4) was experimentally confirmed by rheological analyses, where a significant decrease in the complex viscosity is observed up to 96 h. The changes in the surface wettability and surface energy observed by the contact angle formed by drops of water, observed with 6 h of UV-C photodegradation, can be a result of PP oxidation, as shown in stages (2) and (6).

Between 96 and 384 h of UV-C exposure, two possible pathways may be hypothesized:

- The low molar mass chains formed in the previous stage (4) undergo homolytic bond cleavage upon photon energy absorption, leading to the formation of new radicals (stage 5), which are quickly attacked by molecular oxygen, resulting in the production of other oxidized species (stage 6). At this point, other polypropylene species contribute to the photocatalytic cycle, undergoing hydrogen abstraction by free radical molecules formed in stage (stage 7).
- With the increase in the number of reactive species, i.e., free radicals, from stages (5) and (3), a slight increase in molar mass is observed, between 192 h and 384 h, after the abrupt decrease, between 0 and 96 h. We propose that these species begin recombining, forming branched chains of high molar mass (stage 8). This observation is supported by the increase in complex viscosity (Figure 1) and ^1^H-TD-Low Field NMR (Figure 2), presented in the section *Rheological and ^1^H DQ-TDNMR analyses for the samples*, as well as the decrease in the crystallinity degree, and in the section *Effect of UV-C light on the molecular mobility and microstructure*, and also by the increase in the CH vibration shown in the Raman spectroscopy analysis in Figure S8 in the Supplementary section.

## 5. Conclusions

In this work, the photodegradation of polypropylene under UV-C radiation was studied, focusing on the processes occurring during both shorter and more extended periods of exposure. The rheological analysis revealed a decrease in complex viscosity up to 96 h of exposure, followed by an increase in molar mass and a broadening of the molar mass distribution. In time sweep mode, the samples exhibited unexpected rheological behavior, deviating from what is typically observed in UV-A and UV-B photodegradation of PP, which generally shows a decrease in complex viscosity but maintains time sweep stability. The PP sample exposed to 384 h of UV-C showed instability during the time sweep mode, along with an unusual increase in the complex viscosity. The fraction of unconstrained molecular segments (*f*_d_) and the corresponding relaxation time (*T*_2_) increased for samples exposed for 384 h, supporting the hypothesis of branching formation during long-term exposure. Chemical changes were monitored using FTIR-ATR, contact angle measurements, and surface energy analysis. The methyl index, contact angle, and surface energy proved reliable for detecting polypropylene’s early stages of UV-C-induced photodegradation. Although the carbonyl index is widely used to assess polypropylene photodegradation, this study found it less sensitive than the methyl index in detecting early changes. While the formation of cracks is typically observed in such studies, SEM images in this case showed no crack formation, possibly due to the maximum degree of crystallinity in the studied PP.

Data from ^1^H-TD-NMR suggested that the observed increase in viscosity after 384 h of UV-C exposure may be attributed to chain branching rather than crosslinking. This hypothesis was further supported by DSC analysis.

Finally, these findings underscore the high energy of UV-C radiation and help us propose a reaction pathway for polypropylene photodegradation at this wavelength: chain scission at shorter exposure times and chain branching at longer exposure times. This study yielded significant findings, particularly in addressing the early onset of degradation, a highly critical aspect for specific applications, most notably in the medical field. Moreover, it highlighted the analytical techniques that are most sensitive in identifying such initial changes.

## Figures and Tables

**Table 1:** Parameters extracted from fitting the $I_{nDFMSE}\;vs.\;T\;$ curves using Eq (III).

Sample	$T_{I}({}^{\circ}C)$	$T_{II}({}^{\circ}C)$	$T_{III}({}^{\circ}C)$	$\sigma_{I}({}^{\circ}C)$	$\sigma_{II}({}^{\circ}C)$	$\sigma_{III}({}^{\circ}C)$	$f_{I}$	$f_{II}$	$f_{III}$
PP 0 h	54 ± 3	128 ± 3	167 ± 1	14 ± 1	13 ± 1	3.9 ± 0.5	0.39 ± 0.02	0.25 ± 0.05	0.36 ± 0.02
PP 6 h	54 ± 2	131 ± 5	175 ± 1	14 ± 1	12 ± 2	5 ± 1	0.42 ± 0.02	0.23 ± 0.05	0.35 ± 0.03
PP 12 h	52 ± 2	131 ± 4	177 ± 2	13 ± 1	16 ± 2	6 ± 1	0.40 ± 0.02	0.25 ± 0.05	0.37 ± 0.03
PP 96 h	52 ± 4	133 ± 5	165 ± 2	16 ± 1	13 ± 2	4.5 ± 0.5	0.38 ± 0.02	0.25 ± 0.05	0.38 ± 0.03
PP 192 h	53 ± 2	130 ± 5	157 ± 2	14 ± 1	15 ± 2	3.0 ± 0.5	0.37 ± 0.02	0.26 ± 0.05	0.38 ± 0.03
PP 384 h	59 ± 2	128 ± 4	151 ± 2	14 ± 1	15 ± 2	3.0 ± 0.5	0.40 ± 0.02	0.26 ± 0.05	0.33 ± 0.05

## Data Availability

The original contributions presented in this study are included in the article/Supplementary Material. Further inquiries can be directed to the corresponding author.

## Supplementary Materials

The following supporting information can be downloaded at: https://www.mdpi.com/article/10.3390/polym17202727/s1. Details of the ^1^H TD-NMR technique, UV-C chamber assembly schema, and supporting analysis, such as Raman, DSC, FTIR, and MEV image cross-section of the samples can be found at ‘MDPI-Polymers-Supplementary-material-UV-C.docx’. Figure S1: Schematic illustration of the UV-C chamber employed in the work; Figure S2: G′(ω) and G″(ω) for all PP samples after UV-C exposure; Figure S3: DSC thermogram for the second heating; Figure S4: FTIR spectra of all samples are highlighted in the carbonyl peak; Figure S5: SEM images with magnifications of 20,000× for samples with 12 h, 96 h, and 192 h of exposure time; Figure S6: Cross-section SEM with magnifications 1205× images for samples with 0 h and 384 h of exposure time; Figure S7. PP films with 0 and after 192 and 384 h under UV-C radiation; Figure S8: Raman for samples with 0 h, 96 h, and 384 h under UV-C (decrease in CH_3_, CH bands). Table S1: Wavenumber vibration types and assignment of FTIR peaks for polypropylene; Table S2: Melting Temperature and Degree of Crystallinity for the 2nd heating, and Degree of Crystallinity for the 1st heating values.

## Abbreviations

The following abbreviations are used in this manuscript:

PP	Polypropylene
^1^H TD-NMR	^1^H Time Domain Nuclear Magnetic Resonance
FTIR-ATR	Attenuated Total Reflectance Fourier Transform Infrared spectroscopy
DSC	Differential scanning calorimetry
SEM	Scanning Electron Microscopy
$I_{DQ}$	^1^H nuclei spins
G′(ω)	Storage modulus
G″(ω)	Loss modulus
MI	Methyl index
CI	Carbonyl index
MM	Molar mass
MMD	Molar mass distribution
*f*_d_	Fraction of unconstrained molecular segments
*T*_2_	Relaxation time NMR
DF-MSE	Dipolar Filtered Magic Sandwich Echo
