# Supplementary material for: 1H Time Domain Nuclear Magnetic Resonance and Oscillatory Rheology as a Tool for Uncovering the Impact of UV-C Radiation on Polypropylene
Source: Polymers (Basel). 2025 Oct 11;17(20):2727. doi: 10.3390/polym17202727 (PMC12567356; doi:10.3390/polym17202727)
Supplement: Supplementary file 1 [file polymers-17-02727-s001.zip › polymers-3846166-supplementary.pdf]

SUPPLEMENTARY MATERIAL

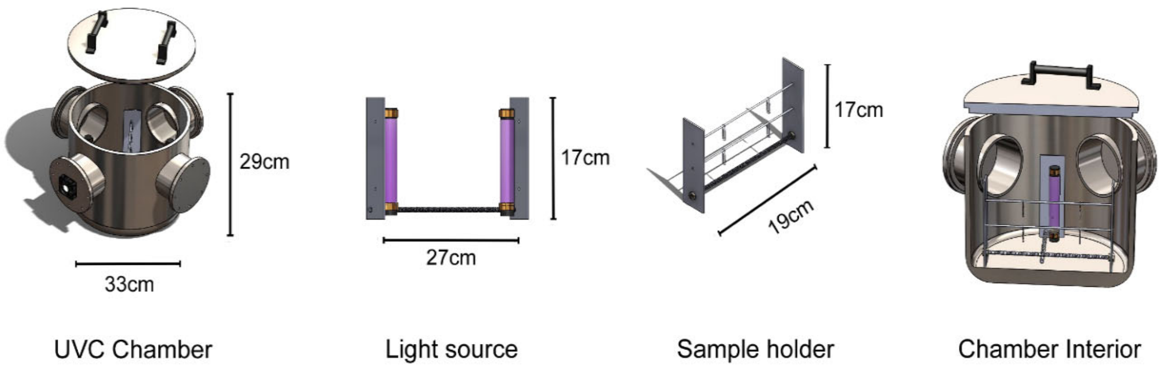

Figure S1. Schematic illustration of the UV-C chamber employed in the work.

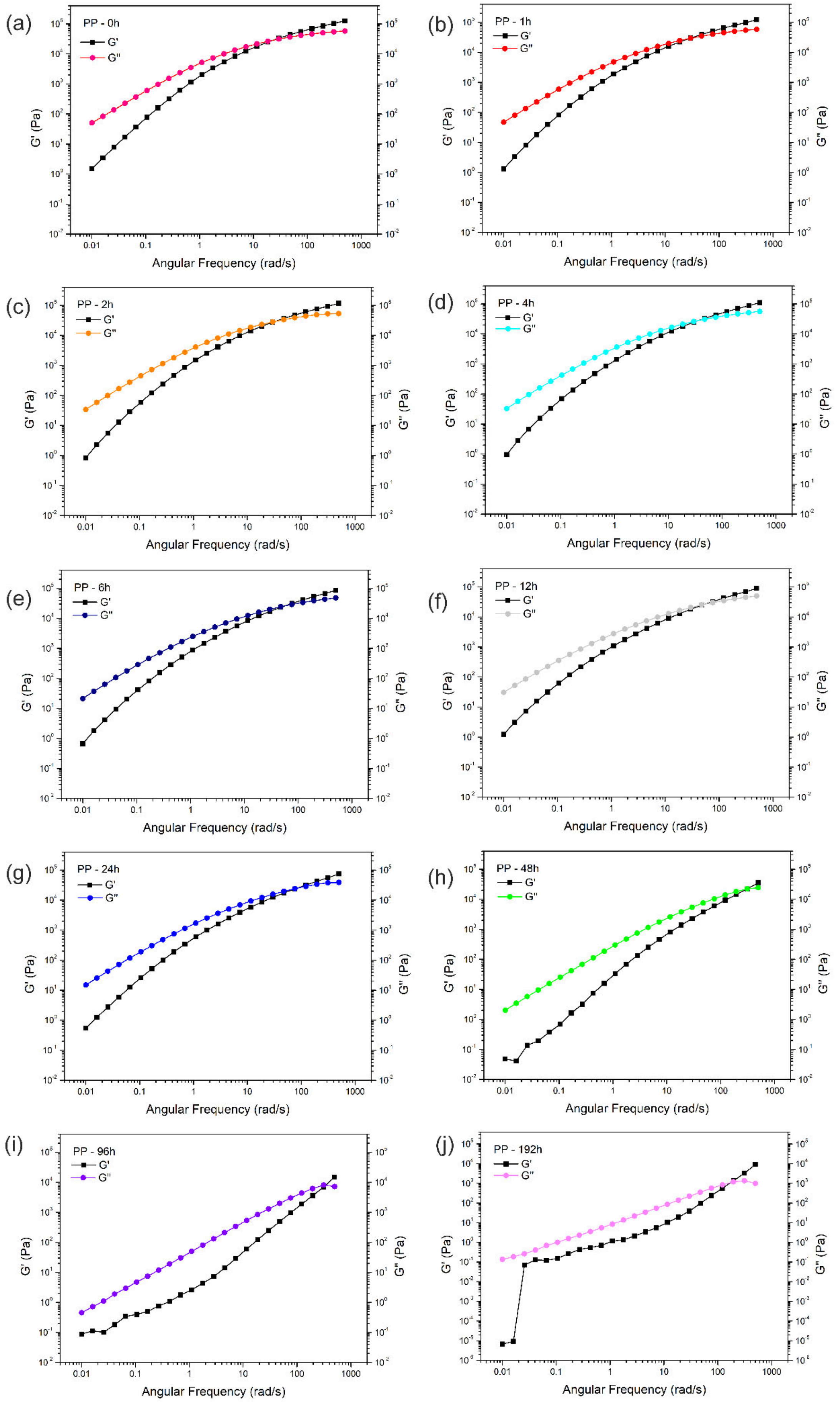
(a)
PP - 0h
G'
G"
G' (Pa)
Angular Frequency (rad/s)
(b)
PP - 1h
G'
G"
G' (Pa)
G" (Pa)
Angular Frequency (rad/s)
(c)
PP - 2h
G'
G"
G' (Pa)
G" (Pa)
Angular Frequency (rad/s)
(d)
PP - 4h
G'
G"
G' (Pa)
G" (Pa)
Angular Frequency (rad/s)
(e)
PP - 6h
G'
G"
G' (Pa)
G" (Pa)
Angular Frequency (rad/s)
(f)
PP - 12h
G'
G"
G' (Pa)
G" (Pa)
Angular Frequency (rad/s)
(g)
PP - 24h
G'
G"
G' (Pa)
G" (Pa)
Angular Frequency (rad/s)
(h)
PP - 48h
G'
G"
G' (Pa)
G" (Pa)
Angular Frequency (rad/s)
(i)
PP - 96h
G'
G"
G' (Pa)
G" (Pa)
Angular Frequency (rad/s)
(j)
PP - 192h
G'
G"
G' (Pa)
G" (Pa)
Angular Frequency (rad/s)

Figure S2. G′(ω) and G″(ω) for all PP samples after UV-C exposure.

Table S1. Melting Temperature and Degree of Crystallinity for the 2nd heating, and Degree of Crystallinity for the 1st heating values.

Time (h)	Melting Temp. Tm - 2nd heating (°C)	Degree of Crystallinity - 2nd heating (%)	Degree of Crystallinity - 1st heating (%)
**0**	163.25 ± 0.21	37.36 ± 1.47	32.59 ± 1.15
**1**	163.10 ± 0.14	45.91 ± 0.09	39.37 ± 0.43
**2**	162.70 ± 0.25	43.67 ± 4.20	37.25 ± 3.51
**4**	162.7 ± 0.42	41.11 ± 3.92	35.22 ± 2.36
**6**	162.80 ± 0.30	43.15 ± 2.12	36.83 ± 1.03
**12**	163.40 ± 0.63	39.91 ± 0.37	35.70 ± 2.13
**24**	163.45 ± 0.63	41.52 ± 0.04	38.79 ± 1.74
**48**	161.59 ± 0.14	42.08 ± 0.66	37.39 ± 1.18
**96**	157.35 ± 1.90	48.38 ± 3.52	42.97 ± 0.50
**192**	153.95 ± 1.20	45.15 ± 0.15	39.45 ± 0.09
**384**	147.01 ± 0.14	31.79 ± 3.45	31.07 ± 5.79

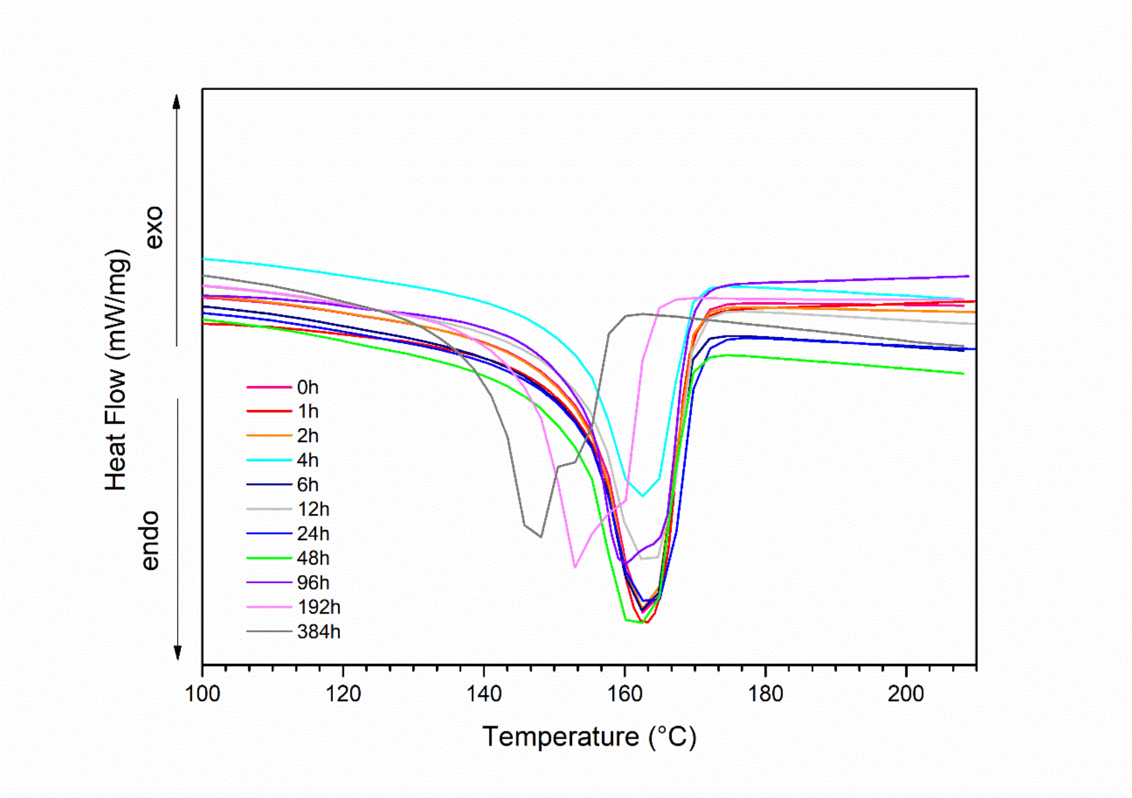

Figure S3. DSC thermogram for the second heating

Table S2. Wavenumber vibration types and assignment of FTIR peaks for polypropylene.

**Wavenumber (cm^{-1})**	**Vibration type**	**Assignment**
2955	Asymmetrical stretching	CH_3
2920	Asymmetrical stretching	CH_2
2869	Stretching	CH_3
	Symmetrical stretching	CH_2
2840	Symmetrical stretching	CH_2
1456	Asymmetrical bending	CH_3
	Bending	CH_2
1375	Symmetrical stretching	CH_3
	Wagging	CH_2
	Bending	CH
	Stretching	C-C
1170	Stretching	C-C
	Rocking	CH_3
	Bending	CH
1153 (shoulder)	Wagging	C-H
	Rocking	CH_3
1001	Rocking	CH_3
976	Rocking	CH_3
	Stretching	C-C
898	-	CH_2
841	Rocking	C-H
805	Stretching	C – C

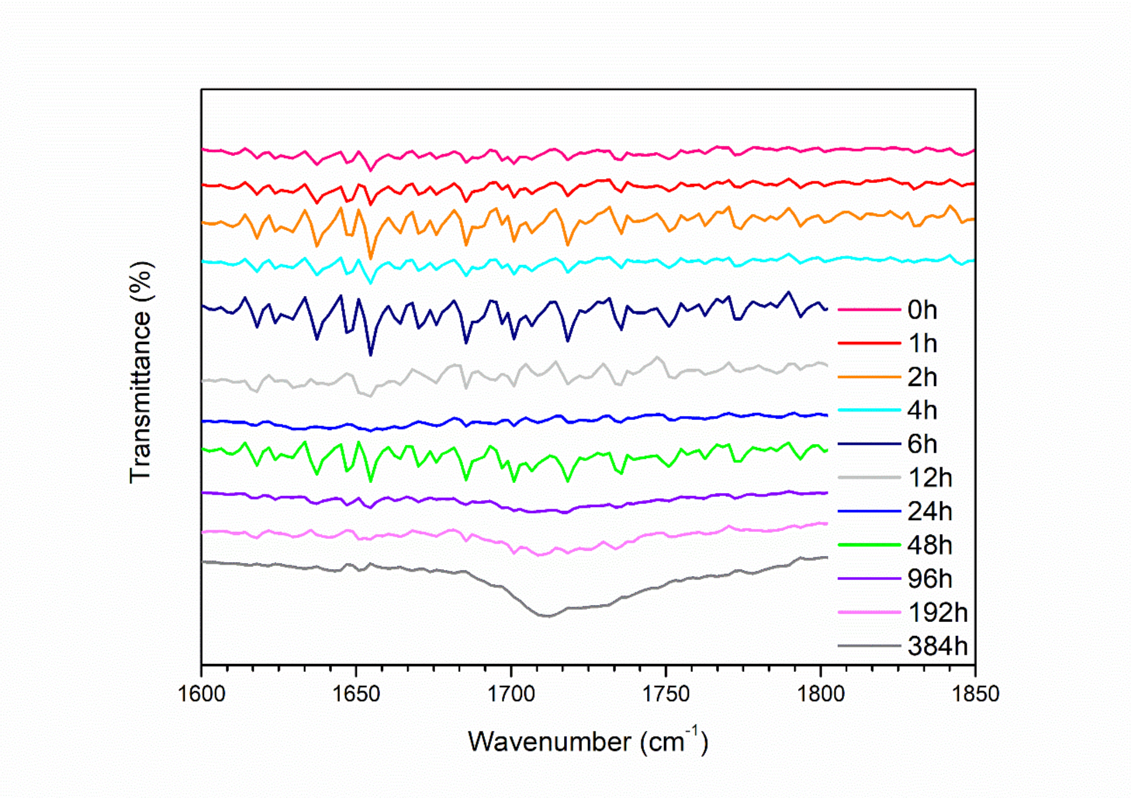

Figure S4. FTIR spectra of all samples are highlighted in the carbonyl peak.

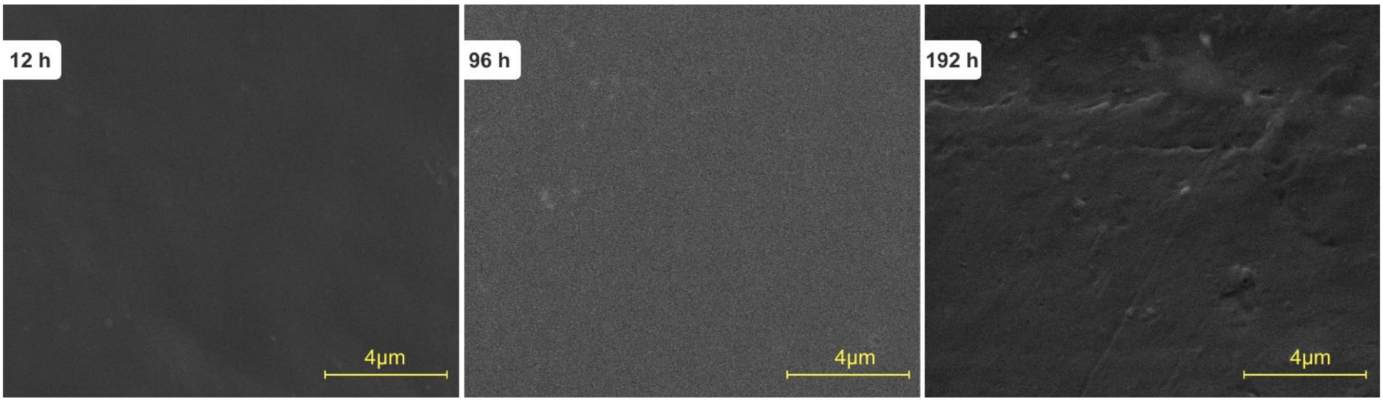

Figure S5. SEM images with magnifications of 20,000 × for samples with 12 h, 96 h, and 192 h of exposure time.

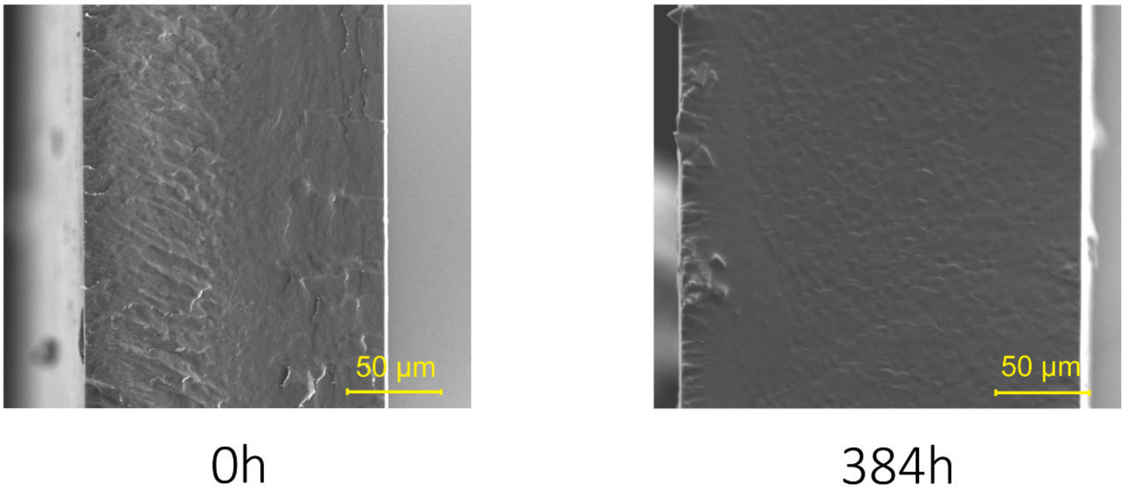

Figure S6. Cross-section SEM with magnifications 1,205 × images for samples with 0h and 384h of exposure time.

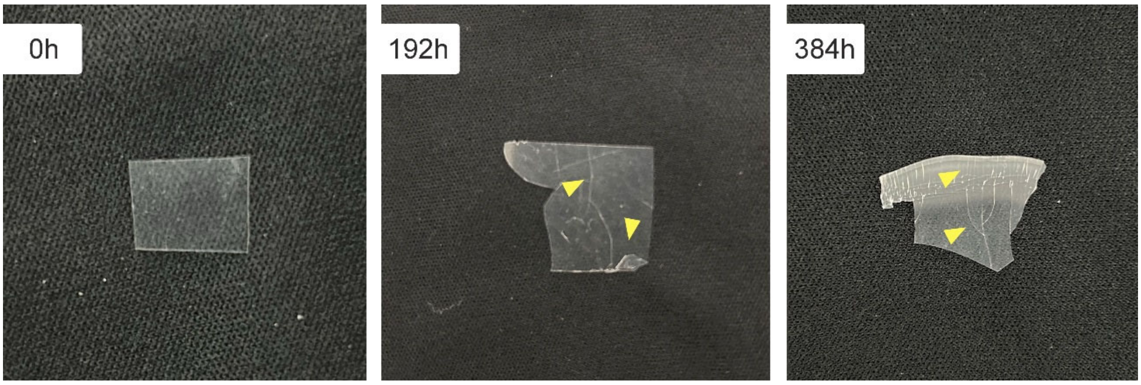

Figure S7. PP films with 0 and after 192 and 384 hours under UV-C radiation.

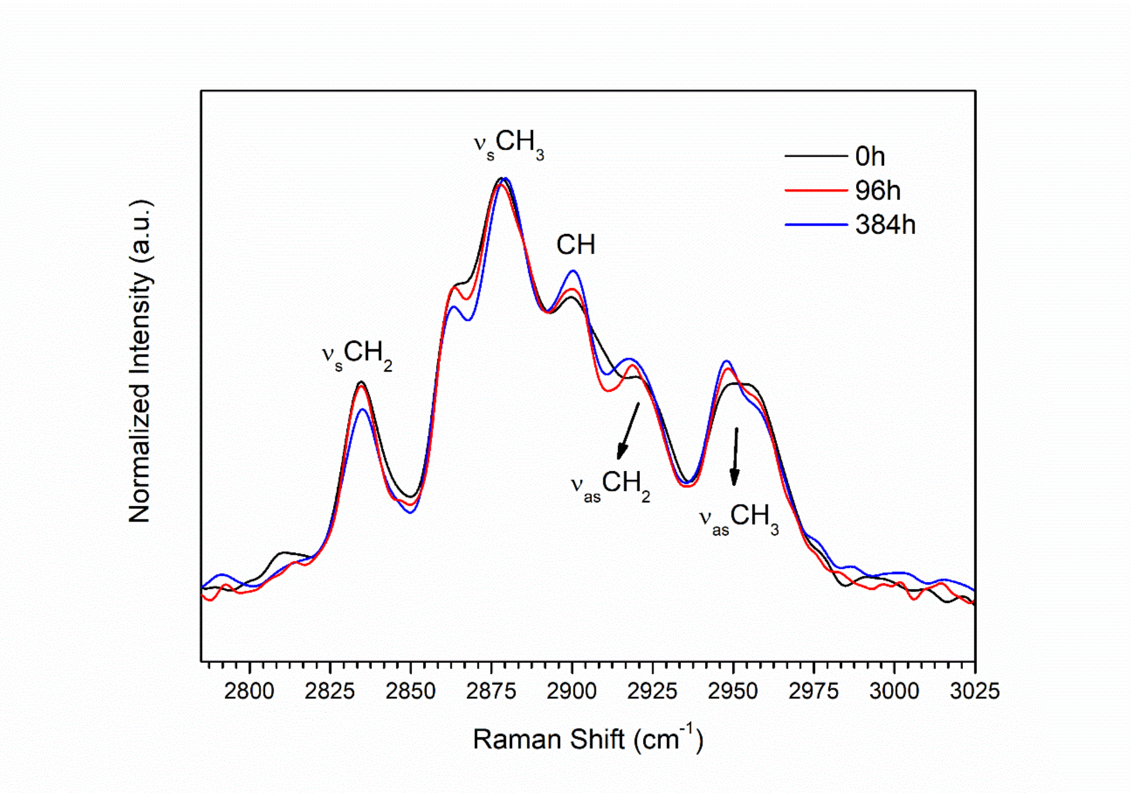

Figure S8. Raman for samples with 0h, 96h, and 384h under UV-C (decrease in CH_3, CH bands)

Micro Raman analysis was performed using a Horiba Jobin-Yvon iHR550 spectrometer with laser excitation at 633 nm. All spectra were collected in a backscattering geometry using a 50X microscope objective.

Figure S4 illustrates the Raman spectrum for the samples with 0, 96, and 384 hours under UV-C. With the increase in exposure time, we can see an increase in the CH vibration, indicating an increase in C-H bonds. It is also possible to see a decrease in the symmetrical vibration of CH_2 bonds [1].

Rheological concepts

Rheology analysis is a technique sensitive to variations in changes in molar mass and in the molar mass distribution. Following the equation [2]:

$$\eta_0 = K[MM]^a$$

Where, η_0 is the zero shear viscosity, MM is the molar mass, and K and a are constants and a assume values from 3.4 to 3.5, which is the slope when the curve $\text{Log}\,(\eta_0) \times \text{Log}\,(MM)$reaches the “critical molar mass value entanglement”, as well as the use of the Cox-Merz rule, which can be expressed as [2,3]:

$$\eta(\dot{\gamma}) = |\eta^*\ (\omega)|$$

When $\dot{\gamma} = \omega$, thus, we can use the zero-shear viscosity from the complex viscosity curve to evaluate the effect of UV-C on the polymer molar mass.

The values of the cross-over point, i.e., when storage modulus $G''(\omega)$ and loss modulus $G''(\omega)$ are the same, can be used to understand changes in the molar mass and in the molar mass distribution [3–5]. Horizontally, the position of this point depends on the polymer's average molar mass, i.e., the crossover point moves towards regions with high frequencies for low-molar-mass polymers. Vertically, this position on the polymer's molar mass distribution, i.e., the cross-over moves to low frequencies when the polymer has a high molar mass distribution.

The use of rheology to understand the effect of UV light on polymers is already established in the literature [6,7] and is also employed in studies to identify the effect of degradation in recycled materials [4,8,9], a method widely used.

^{1}H TD-NMR – technique details

The alteration in molar mass with the photodegradation can be evaluated using a class of ^{1}H TD-NMR experiments named ^{1}H double-quantum time domain NMR (^{1}H DQ-TDNMR) [10]. ^{1}H DQ-TDNMR is well known for its ability to probe structural and dynamical features in polymer networks such as elastomers and polymer gels [11]. Notably, ^{1}H DQ-TDNMR can bring information about the presence of chemical and physical constraints and also about the fraction of free mobile (not constrained) chains [12,13]. .^{1}H DQ-TDNMR pulse sequences are designed to detect a signal intensity related to double quantum coherences between ^{1}H nuclei (I_{DQ}), induced by the presence of ^{1}H-^{1}H dipolar magnetic coupling. This coupling arises only when there are chemical and/or physical motional constraints within polymer chains, rendering the double quantum intensity I_{DQ} nonzero only in the presence of such constraints.

In a crosslinked/entanglement polymer melt, the magnitude of the ^{1}H-^{1}H dipolar coupling is strongly reduced by the overall motion of the chains, remaining only a residual coupling, which is proportional to the cube of the distance between crosslink/entanglement points. In other words, the magnitude of this residual dipolar coupling is proportional to the density of crosslinked/entangled chains. ^{1}H DQ-TDNMR are usually performed as a function of an experimentally set evolution period τ_{DQ} , so a $I_{DQ}.vs.\tau_{DQ}$ curve, usually known as DQ build-up curves, is obtained. DQ build-up curves show an initial increase due to the creation of the DQ coherences, followed by an exponential type of attenuation with a decay rate given by the inverse of the transverse

spin relaxation time T_2 of the dipolar coupled spins. In addition, the initial slope is proportional to the residual of ^1H-^1H dipolar coupling, so it is directly proportional to the density of crosslinks/entanglements.

A simple adjustment in pulse phases of the ^1H DQ-TDNMR experiments yields another signal, with intensity associated with all ^1H spins in the sample (I_{REF}). The decay of the I_{REF} intensity as a function of τ_{DQ} is also an exponential type of attenuation rate is the inverse of the transverse spin relaxation time T_2, but including all segments. Thus, because dipolar coupled spins have shorter transverse relaxation times than non-coupled ones, at longer τ_{DQ} times the decay of the $I_{REF}.vs.\tau_{DQ}$ curve is only due to segments which do not experience dynamical constraints. Essentially, the long τ_{DQ} tail of the $I_{REF}.vs.\tau_{DQ}$ is proportional to the amount of polymer chains devoid of mobility constraints, encompassing network defects like pendant chains (dangling ends), chain loops, free chains trapped within the network, and/or particulate fillers in a crosslinked polymer network [12,13]. The fraction of network defects and the T_2 relaxation times can be quantitatively obtained by subtracting the I_{REF} and I_{DQ} to eliminate the contribution of crosslinked/entangled chains and fitting the extended $2\tau_{DQ}$ tail of the I_{REF} - I_{DQ} curve with an exponential function $f_d e^{-\frac{2\tau_{DQ}}{T_2}}$, so the decay time and amplitude of the fitting curves provide estimations of the T_2 relaxation time and the fraction of network defects.

For accurate results, the polymer chains need to exhibit high mobility, so the motion constraints will be only due to crosslinked/entangled chains. In a semi-crystalline polymer like PP, this means that all the experiments need to be done in the molten state.

Another TD-NMR experiment used here is known as Dipolar Filtered Magic Sandwich Echo (DF-MSE) [14]. Simply put, this experiment yields a normalized signal intensity I_{nDFMSE}, which accounts exclusively for molecular segments that undergo molecular motions with rates higher than the ^1H-^1H magnetic dipolar coupling, typically in the order of 50 kHz. Thus, by monitoring the I_{nDFMSE} as a function of temperature, it becomes possible to directly detect the onset temperature of molecular mobility processes through the increase in I_{nDFMSE}. For instance, if we start at a temperature where molecular motions are minimum in the sample and then increase the temperature, an upturn in intensity is observed in the $I_{nDFMSE}.vs.T$ plot when certain molecular segments become mobile. Hence, this method can detect the onset temperature of specific segmental motions by tracking I_{nDFMSE} as a function of temperature.

References

1. Fitaroni, L.B.; Cacuro, T.A.; Costa, C.A.R.; Lanzoni, E.M.; Galante, D.; Araujo, J.R.; Homem, M.G.P.; Waldman, W.R.; Cruz, S.A. Polymeric Nanowrinkles: Surface Modification of Polypropylene Films in the VUV Energy Range. *J. Mater. Sci.* **2021**, *56*, 9532–9543, doi:10.1007/s10853-021-05879-1.
2. Dealy, J.M..; Wissbrun, K.F. *Melt Rheology and Its Role in Plastics Processing*; Springer Netherlands: Dordrecht, 1990; Vol. 3; ISBN 978-0-7923-5886-2.
3. Bretas, R.E.S.; D'Avila, M.A. *Reologia de Polímeros Fundidos*; 2nd ed.; EdUFSCar: São Carlos, 2005; ISBN 85-7600-048-2.
4. Cruz, S.A.; Zanin, M. Evaluation and Identification of Degradative Processes in Post-Consumer Recycled High-Density Polyethylene. *Polym. Degrad. Stab.* **2003**, *80*, 31–37, doi:10.1016/S0141-3910(02)00379-8.
5. Mezger, T.G. *Applied Rheology With Joe Flow on Rheology Road*; 9th ed.; Anton Paar GmbH: Austria, 2021; ISBN 978-3-9504016-0-8.
6. Mylläri, V.; Ruoko, T.P.; Syrjälä, S. A Comparison of Rheology and FTIR in the Study of Polypropylene and Polystyrene Photodegradation. *J. Appl. Polym. Sci.* **2015**, *132*, 1–6, doi:10.1002/app.42246.
7. Rouillon, C.; Bussiere, P.-O.; Desnoux, E.; Collin, S.; Vial, C.; Therias, S.; Gardette, J.-L. Is Carbonyl Index a Quantitative Probe to Monitor Polypropylene Photodegradation? *Polym. Degrad. Stab.* **2016**, *128*, 200–208, doi:10.1016/j.polymdegradstab.2015.12.011.
8. Oliveira, I.M.; Gimenez, J.C.F.; Xavier, G.T.M.; Ferreira, M.A.B.; Silva, C.M.P.; Camargo, E.R.; Cruz, S.A. Recycling ABS from WEEE with Peroxo-Modified Surface of Titanium Dioxide Particles: Alteration on Antistatic and Degradation Properties. *J. Polym. Environ.* **2023**, doi:10.1007/s10924-023-03021-7.
9. Freitas, F.L.S.; Chinellato, A.C.; Cruz, S.A. Molar Mass Alteration During Post-Consumer PET Recycling Using Polycarbodiimide-Based Additive. *J. Polym. Environ.* **2021**, *29*, 734–744, doi:10.1007/s10924-020-01896-4.
10. Baum, J.; Munowitz, M.; Garroway, A.N.; Pines, A. Multiple-Quantum Dynamics in Solid State NMR. *J. Chem. Phys.* **1985**, *83*, 2015–2025, doi:10.1063/1.449344.
11. Saalwächter, K.; Herrero, B.; López-Manchado, M.A. Chain Order and Cross-Link Density of Elastomers As Investigated by Proton Multiple-Quantum NMR. *Macromolecules* **2005**, *38*, 9650–9660, doi:10.1021/ma051238g.
12. Chassé, W.; Lang, M.; Sommer, J.U.; Saalwächter, K. Erratum: Cross-Link Density Estimation of PDMS Networks with Precise Consideration of Networks Defects (Macromolecules (2012) 45:2 (899) DOI: 10.1021/Ma202030z). *Macromolecules* **2015**, *48*, 1267–1268, doi:10.1021/acs.macromol.5b00236.
13. Chassé, W.; Lang, M.; Sommer, J.-U.; Saalwächter, K. Correction to Cross-Link Density Estimation of PDMS Networks with Precise Consideration of Networks Defects. *Macromolecules* **2015**, *48*, 1267–1268, doi:10.1021/acs.macromol.5b00236.
14. Filgueiras, J.G.; da Silva, U.B.; Paro, G.; D'Eurydice, M.N.; Cobo, M.F.; DeAzevedo, E.R. Dipolar Filtered Magic-Sandwich-Echoes as a Tool for Probing Molecular Motions Using Time Domain NMR. *J. Magn. Reson.* **2017**, *285*, 47–54, doi:10.1016/j.jmr.2017.10.008.
